# Eight-week flexi-bar vibration exercise improves cardiovascular function accompanied by altered gut microbiota and metabolites in middle-aged and elderly women: a randomized controlled trial

**DOI:** 10.3389/fcvm.2026.1925079

**Published:** 2026-10-27

**Authors:** Dongdong Gao, Weiji Deng, Min Hu, Younian Mai, Guangnan Liang, Jiarui Wu, Yanying Wu, Jinghui Guo, Junhao Huang

**Affiliations:** 1 Guangdong Provincial Key Laboratory of Physical Activity and Health Promotion, Guangzhou Sport University, Guangzhou, Guangdong, China; 2 School of Medicine, The Chinese University of Hong Kong, Shenzhen, Guangdong, China; 3 Dr. Neher’s Biophysics Laboratory for Innovative Drug Discovery, State Key Laboratory of Quality Research in Chinese Medicine, Macau University of Science and Technology, Macao SAR, China

**Keywords:** arterial stiffness, cardiovascular function, flexi-bar vibration exercise, gut microbiotal, SCFAs

## Abstract

**Aims:**

This study aimed to investigate the impact of 8-week FE on cardiovascular health in middle-aged and elderly women and explore correlational associations involving gut microbiota and its metabolites.

**Methods:**

Thirty middle-aged and elderly women were randomly divided into FE group and control group. Body composition parameters were assessed using a body composition analyzer. cfPWV and baPWV were detected to assess central and systemic arterial stiffness, respectively. To evaluate central hemodynamic parameters and cardiac autonomic function, HR, augmentation index, SEVR, and HRV were measured. Biochemical factors in serum were detected by ELISA. Gut microbiota and SCFAs levels in Fecal samples were assessed by 16S rRNA gene sequencing and GC-MS.

**Results:**

FE helped preserve water, inorganic salts, muscle mass, and fat-free mass, while prevented the accumulation of body weight and body fat. FE also significantly decreased HR, central systolic and diastolic blood pressure, cfPWV and R-baPWV. The HRV analysis demonstrated that FE increased SDNN and NN50 (time-domain indices) and high-frequency band, while decreasing low-frequency band. Moreover, FE significantly up-regulated the serum eNOS level and down-regulated ICAM1 level. Meanwhile, FE increased *Bacteroidota* abundance and decreased *Firmicutes* abundance, alongside elevated fecal acetic acid, propionic acid, and caproic acid in fecal samples.

**Conclusions:**

8-week FE improves cardiovascular function in middle-aged and elderly women by enhancing parasympathetic activity, reducing sympathetic overactivity, and mitigating arterial stiffness. Parallel shifts in gut microbiota composition and elevated SCFAs were observed following FE intervention, highlighting FE as an effective intervention for cardiovascular health in this population.

**Clinical Trial Registration:**

http://www.chictr.org.cn/, identifier ChiCTR2000041245.

## Introduction

According to an estimated data from “Report on Cardiovascular Health and Diseases in China 2021”, approximately 330 million individuals are suffering from cardiovascular disease (CVD) which accounts for more than 40% of all deaths in China (1, 2). The cardiovascular health-related factors including age, physical activity level, diet, tobacco use, metabolic syndrome, psychological factors, etc (3). Aging, as a natural and inevitable part of human life, has been predicted to become one of the most important global health challenges in the latter half of this century (4), and a powerful motivator of increasing CVD incidence and prevalence (5, 6). Notably, middle-aged and elderly women face a sharp increase in CVD risk post-menopause, attributed to dramatic hormonal changes (7, 8). Cardiovascular risk factors also lead to earlier, faster symptom progression in this group than in men of the same age (9). However, most prior studies on exercise and cardiovascular health have focused on men or mixed-gender populations, resulting in a lack of gender-specific evidence for middle-aged and elderly women.

Over the past decades, robust scientific evidence supported the benefits of regular exercise on delaying the aging process, and reducing CVD risk by reducing resting blood pressure (BP), risk of metabolic syndrome, and blood glucose level, improving body composition, regulating insulin secretion, etc (10–12). Despite these well-documented benefits, the underlying mechanisms linking exercise to cardiovascular protection remain incompletely understood. Recently, gut-heart axis has been considered as a new target for the treatment of CVD (13). Our previous study has demonstrated that *R. lituseburensis JCM1404* supplementation could ameliorate endothelial function via altering lipid metabolisms in obese rats (14), highlighting the role of gut microbiota in vascular health. Beyond this, a growing body of evidence has established a correlation between exercise-induced improvements in cardiovascular health and changes in gut microbiota composition (15–18). Crucially, gut microbiota exerts regulatory effects on cardiovascular function primarily through its metabolites short-chain fatty acids (SCFAs), which have been explicitly shown to mediate the anti-atherosclerotic effects of endurance exercise (19). Even so, the specific roles of gut microbiota and its derived metabolites in modulating exercise-driven cardiovascular benefits remain to be clearly defined.

Flexi-bar vibration exercise (FE) is a unique local vibration motion form, relying on aerobic energy as its predominant energy supply (20). It produces vibrations at a frequency of approximately 5 Hz, equivalent to 300 oscillations per minute. A recent research showed that FE with different postures and amplitudes impose no extra threats to acute cardiovascular stress (21), indicating FE is a safe exercise modality for middle-aged and elderly people. However, few studies have explored the potential beneficial effects of FE on cardiovascular function, gut microbiota, and its metabolites. The present study therefore aimed to investigate the influence of 8-week FE intervention on cardiovascular function in middle-aged and elderly women and reveal the potential underlying mechanisms, with a specific focus on the role of gut microbiota and its metabolites correlated with FE's cardiovascular effects.

## Materials and methods

### Participants

The eligibility criteria of participants enrolled in the present study were described as follow: middle-aged and elderly women whose age were 45 years old and above; physically health and have no regular exercise (fewer than 3 times per week, and less than 20 min each time), voluntarily signed the written informed consent prior to participation. The exclusion criteria were described as follow: spasmodic disease during the study; intake of oral antibiotics, probiotic supplements, prebiotic preparations, or proton pump inhibitors (PPIs) within three months before enrollment; voluntary withdrawal from the trial; unable to complete the 8-week FE training as required. All participants were required to maintain their habitual daily diet without substantial dietary adjustments during the 8-week intervention period. The present study was conducted according to declaration of Helsinki and approved by the Ethics Committee of Guangzhou Sport University (No: 2020LCLL-008), registered in the Chinese Clinical Trial Registry (http://www.chictr.org.cn/) on September 22, 2020 (No: ChiCTR2000041245). All participants provided written informed consent prior to enrollment, and the study was conducted in accordance with the Declaration of Helsinki.

### Study design

The present trial was a randomized, parallel-group controlled trial. The study was conducted from October 19, 2020 to December 19, 2020. Sample size was *a priori* calculated via G*Power 3.1 software based the pre-specified primary outcomes: arterial stiffness indices cfPWV and baPWV. For one-way between-groups ANOVA with two study arms, we set *α* = 0.05, 80% statistical power, and a large effect size (Cohen's *f* = 0.64), with a minimum of 10 participants per group required. Thirty-five participants were recruited at the beginning of this study. Thereafter, 5 participants were excluded from the study due to their own reasons. Finally, 30 participants who meeting the elibility criteria were randomly divided into the FE group and the control group in average. A simple random sequence was generated by an independent third-party investigator not involved in recruitment, assessments, or data analysis. Allocation concealment was implemented using sequentially-numbered, sealed, opaque envelopes; recruitment staff were separated from the researcher conducting group assignment and had no access to the random sequence before eligibility confirmation. Four participants assigned to the FE group withdrew before completing the the 8-week intervention, as they failed to finish all physical examinations or exercise session. All unexpected physical discomfort and exercise-related adverse reactions were documented throughout the 8-week training program for *post-hoc* safety evaluation. All randomized participants, who completed the 8-week intervention, fulfilled the pre-specified compliance criteria and maintained full attendance over the entire intervention period. No severe cardiovascular or musculoskeletal adverse events were reported during the trial. All outcome assessors responsible for physiological measurements and laboratory staff processing fecal and blood samples were fully blinded to participants' group assignments throughout data collection, testing and statistical analysis. Besides, only participants with complete paired baseline and post-intervention outcome data were included in the final statistical analyses (FE group: *n* = 11; Control group: *n* = 15).The experimental design for this trial was illustrated in Figure 1. The basic physical conditions of the participants of these two groups were shown in Table 1.

**Figure 1:**
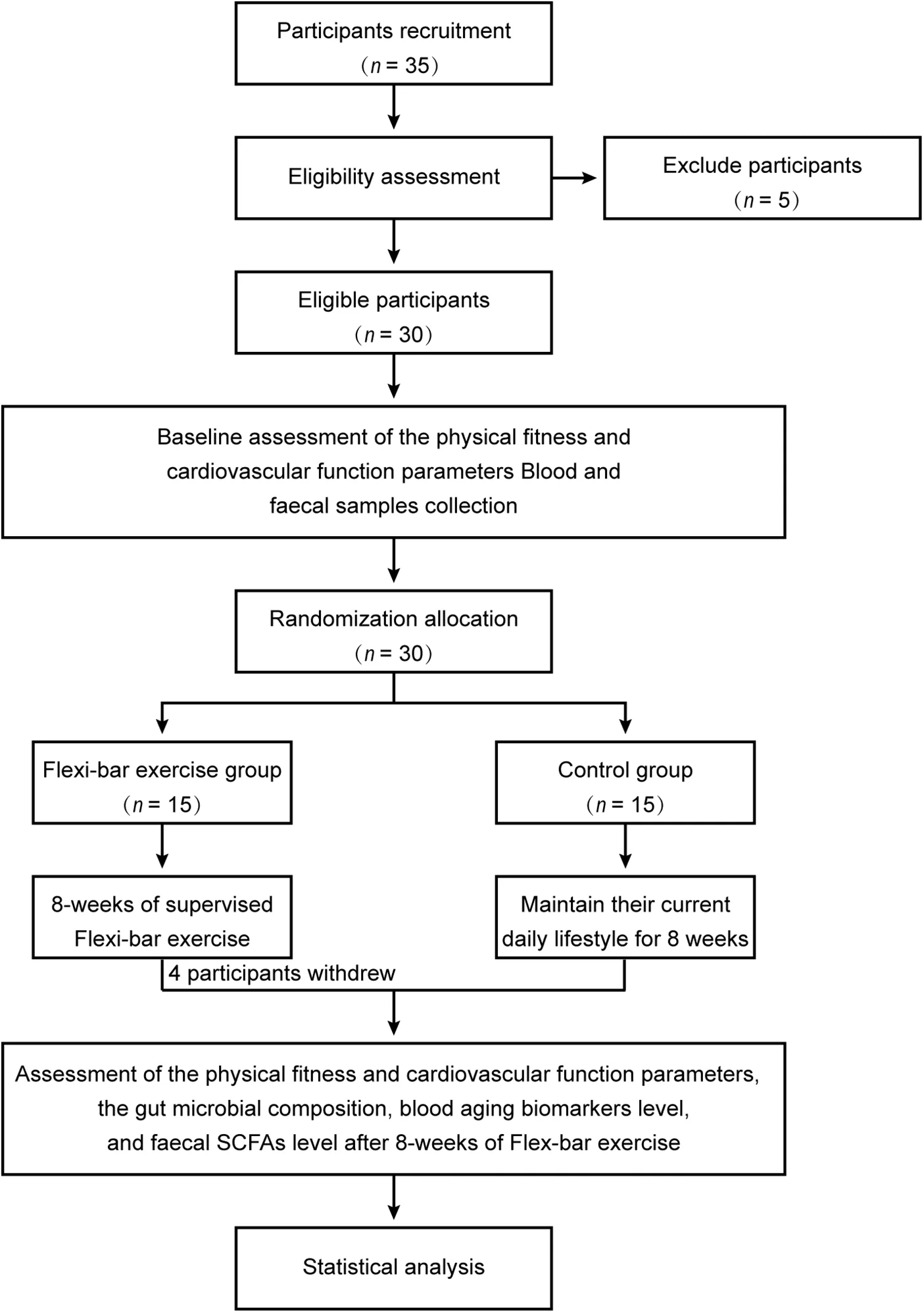
Participant flow-chart of the present trial.

**Table 1:** The physical conditions of the participants at the beginning of the study.

Parameters	Control group	FE group
Age (year)	55.47 ± 5.45	56.53 ± 5.64
Height (cm)	158.53 ± 6.08	158.93 ± 4.79
Weight (kg)	57.02 ± 5.67	55.60 ± 6.22
BMI (kg/m^2^)	22.71 ± 2.16	22.02 ± 1.86
Water content (kg)	27.99 ± 2.85	26.77 ± 2.22
Inorganic salt content (kg)	2.67 ± 0.28	2.60 ± 0.21
Protein content (kg)	7.44 ± 0.72	7.13 ± 0.61
Muscle content (kg)	35.92 ± 3.63	34.35 ± 2.84
Fat mass (kg)	18.59 ± 3.44	18.91 ± 4.18
Fat-free mass (kg)	38.11 ± 3.84	36.24 ± 3.15
Fat mass percentage (%)	32.65 ± 4	33.98 ± 4.85
HR (bpm)	60.64 ± 7.48	61.27 ± 6.65
CSP (mmHg)	119.79 ± 10.2	122.55 ± 17.03
CDP (mmHg)	75.93 ± 6.87	79.73 ± 7.89
cfPWV (m/s)	7.16 ± 0.68	7.3 ± 0.65
L-baPWV (m/s)	11.3 ± 0.82	11.84 ± 1.25
R-baPWV (m/s)	11.18 ± 0.8	11.22 ± 1.19
AIX (%)	37.07 ± 10.81	35.73 ± 9.68
AIX@75 (%)	29.86 ± 9.84	30.55 ± 8.55
HRV (ms)	7.27 ± 1.97	7.25 ± 2.55
RMSSD (ms)	29.19 ± 19.57	32.32 ± 18.43
pNN50 (%)	9.59 ± 18.87	11.98 ± 18.95
SDNN (ms)	35.84 ± 13.84	35.76 ± 12.62
NN50 (count)	23.93 ± 44.28	27.73 ± 40.43
HF (ms^2^)	0.23 ± 0.06	0.24 ± 0.06
LF/HF	1.19 ± 1.24	1.45 ± 1.14
TP (ms^2^)	936.79 ± 787.79	954 ± 809.78

### FE training protocol

The vibration equipment (Flexi-bar®, Flexi-Sports, Germany) employed in the FE training is a glass fiber elastic bar, which is 1.53 m long, 719 g, and 9.5 mm for circumference. Participants grasped the Flexi-bar with both hands and performed dynamic vibration exercises, producing an approximate 5 Hz stimulus. The FE group completed 8 weeks of FE training, 3 times per week and 60 min per time, under the guidance of a qualified expert. The FE training protocol consist of a 5 min warm up performing static stretch followed by dynamic warm up with the Flexi-bar, a 50 min workout performing 10 specific Flexi-bar training model positions (chest, balance abductor, waist, triceps, biceps, core muscle, deep back extensor, shoulder, hips, and lower back) with each position including 3 sets of 60 s vibration and 20 s of rest, an extra 1 min rest interval was scheduled after finishing each of the 10 specific Flexi-bar training positions to switch postures. And a 5 min cool down performing to static stretch of the major muscle groups. The Polar watch was used to monitor the exercise intensity, which was controlled at 50% to 60% of HRmax (HRmax = 220 − age). Participants in the control group were requested to maintain their current daily lifestyle as much as possible and avoid regular exercise during this study.

### Assessment of body composition

The parameters of the body composition, including body weight, body mass index (BMI), fat-free mass, fat mass, fat mass percentage, water content, inorganic salt content, protein content, and muscle content, were assessed using bioelectrical impedance method via the body composition analyzer InBody 370 (InBody, Biospace; Seoul, South Korea) according to the manufacturer's instructions.

### Cardiovascular function measurements

Arterial stiffness and hemodynamic measurements were performed using the SphygmoCor (AtCor Medical, Sydney, NSW, Australia) system. Central arterial stiffness is evaluated by carotid-femoral pulse wave velocity (cfPWV). The central hemodynamic parameters, including heart rate (HR), central systolic blood pressure (CSP), central diastolic blood pressure (CDP), augmentation index of reflected wave (AIx), augmentation index standardized to a heart rate of 75/min (AIx@75), and subendocardial viability ratio (SEVR) were measured. The HRV assessment, measuring both the time and frequency domain indices, was performed for the cardiac autonomic function evaluation. The time-domain indices include the square root of the mean of the sum of squares of differences between adjacent normal to intervals (RMSSD), number of pairs of adjacent NN intervals differing by more than 50 ms (NN50), NN50 count divided by the total number of NN intervals (pNN50), and the standard deviation of all the NN intervals (SDNN). The frequency-domain indices include the total power (TP) in the frequency range (0–0.40 Hz) which was divided into low-frequency power (LF: 0.04–0.15 Hz, modulated mainly by the sympathetic system) and high-frequency power (HF: 0.15–0.40 Hz, modulated by the parasympathetic system). LF/HF is the ratio of LF to HF. In addition, the ABI system (boso ABI-system 100; BOSCH & SOHN, Germany) was employed to measure brachial-ankle pulse wave velocity (baPWV), which is used to evaluate systemic arterial stiffness.

### Enzyme-linked immunosorbent assay (ELISA) measurements

The blood samples were collected in tubes without anticoagulant for serum preparation. Serum was then obtained by centrifugation (1,500 g for 15 min at 22 ℃) and stored at −80 ℃ until further analysis. The contents of the eNOS, VEGF, ICAM-1, VCAM-1, and TNF-α in the serum were detected using ELISA Kits (Cusabio, Biotech. Co., Ltd., Wuhan, China) according to the manufacturer's instructions.

### Fecal sample collection and 16S rDNA sequencing

The fecal samples of the participants were collected in separate sterile fecal collection tubes (5 mL) before and after the FE intervention. The fecal samples were collected, and then placed into the liquid nitrogen immediately for preservation. The detailed fecal total genomic deoxyribonucleic acid (DNA) extracting processing was described as before (22). The V3–V5 hypervariable regions of the bacterial 16S rRNA gene were amplified for sequencing library construction following Illumina's standard 16S metagenomic workflow. Briefly, PCR amplicons were purified via filter plates and quantified using a Qubit® 3.0 fluorometer (Life Invitrogen). Raw sequencing reads generated on the Illumina MiSeq platform were processed in QIIME 2 (version 2019.4) adopting the ASV/DADA2, which included quality filtering, denoising, read merging, and chimera removal. Taxonomic classification of ASV representative sequences was conducted against the SILVA-138 reference database, and *α*- and *β*-diversity metrics were subsequently calculated. Weighted UniFrac-based principal-coordinate analysis (PCoA) was visualized with the R ade4 package. Sequencing quality indicators (raw read counts, Q20/Q30 values, read numbers remaining after filtering-denoising-merging-himera removal) are summarized in Supplementary Table S1.

### Quantitative analysis of short-chain fatty acids (SCFAs) levels

Gas chromatography-mass spectrometry (GC-MS) was used for SCFAs level detection. Fecal samples were prepared and the SCFAs were extracted and was described previously (23). In brief, 50 mg fecal sample was added to a reaction system, which contains 15% phosphoric acid (50 µL), 125 µg/mL internal standard (isohexanoic acid) solution (100 µL) and diethyl ether homogenate (400 µL). After 1 min of reaction, the mixture was centrifuged at 12,000 RPM for 10 min at 4 °C. The supernatant was then collected to a vial for the subsequent GC analysis. SIM was used to collect and quantitatively measure known characteristic ions, and Chromeleon7.0 software was used for integration, and the content was calculated using standard curve. All samples were assayed in duplicate. Baseline and post-intervention specimens from each participant were measured in the same batch to reduce batch variation. Lab technicians were blinded to group assignments during testing. All samples underwent no more than two freeze-thaw cycles to prevent analyte deterioration. All pairwise associations displayed in heatmaps were calculated using Spearman's rank correlation. These correlation analyses are purely exploratory and hypothesis-generating.

### Statistical analysis

Statistical analysis was performed using GraphPad Prism version 7.0 (GraphPad Software, CA, USA). All baseline indicators were balanced between groups (all *P* > 0.05, Table 1). Accordingly, the unpaired *t*-test was used for the assessment of the difference between the FE group and the control group. ANCOVA or repeated-measures mixed-effects models would be statistically preferable for this pre-post trial design, but these approaches were not adopted given our small sample size and post-randomization drop-outs within the FE group. The data was presented as mean and standard deviation (SD). The Benjamini–Hochberg FDR correction was used to control multiple comparison bias. Normality tests validated the applicability of parametric t-tests. Significance was defined as FDR-adjusted *P* < 0.05 (Table 2).

**Table 2:** Statistical comparison of outcome change scores between the two groups.

Parameters	Mean difference	95%.CI	Cohen's *d*	FDR-adjusted
Weight (kg)	−0.68	[−1.12, −0.25]	−1.30	*P* = 0.004
BMI (kg/m^2^)	−0.13	[−0.38, 0.13]	−0.42	*P* = 0.314
Water content (kg)	0.82	[0.33, 1.31]	1.38	*P* = 0.002
Inorganic salt content (kg)	0.13	[0.05, 0.22]	1.37	*P* = 0.002
Protein content (kg)	0.13	[−0.05, 0.31]	0.64	*P* = 0.147
Muscle content (kg)	0.91	[0.28, 1.55]	1.20	*P* = 0.007
Fat mass (kg)	−1.38	[−2.08, −0.68]	−1.65	*P* = 0.001
Fat-free mass (kg)	0.70	[0.09, 1.31]	0.98	*P* = 0.027
Fat mass percentage (%)	−2.09	[−3.20, −0.98]	−1.56	−1.56
HR (bpm)	−5.91	[−9.40, −2.42]	−1.42	*P* = 0.002
CSP (mmHg)	−9.25	[−15.31, −3.20]	−1.29	*P* = 0.004
CDP (mmHg)	−4.55	[−8.89, −0.22]	−0.87	*P* = 0.040
cfPWV (m/s)	−1.09	[−1.64, −0.54]	−1.67	*P* = 0.001
L-baPWV (m/s)	−0.82	[−1.40, −0.23]	−1.15	*P* = 0.008
R-baPWV (m/s)	−0.87	[−1.57, −0.16]	−1.04	*P* = 0.018
AIX (%)	−7.58	[−12.68, −2.47]	−1.27	*P* = 0.005
AIX@75 (%)	−5.85	[−11.26, −0.44]	−0.90	*P* = 0.035
HRV (ms)	1.46	[−0.00, 2.93]	0.84	*P* = 0.050
RMSSD (ms)	−5.80	[−17.79, 6.19]	−0.42	*P* = 0.327
pNN50 (%)	−3.24	[−11.89, 5.41]	−0.32	*P* = 0.446
SDNN (ms)	11.18	[4.28, 18.07]	1.39	*P* = 0.003
NN50 (count)	16.97	[3.71, 30.24]	1.09	*P* = 0.014
HF (ms^2^)	0.15	[0.08, 0.23]	1.74	*P* = 0.001
LF/HF	−0.43	[−0.64, −0.22]	−1.76	*P* = 0.001
TP (ms^2^)	461.00	[−23.56, 945.60]	0.81	*P* = 0.061

## Results

### FE improved body composition

As shown in Figure 2A, 8-week FE mitigated excessive weight gain compared with the control group, rather than inducing absolute weight loss However, there were no significant differences in BMI and protein content changes between the control group and the FE group (Figures 2B,E). Meanwhile, we observed that the water content (Figure 2C), inorganic ion content (Figure 2D), muscle content (Figure 2F), and fat free mass (Figure 2H) significantly increased in the FE group, along with a opposite trend in the control group. Our data also showed that 8-week FE intervention could significantly decrease the fat mass (Figure 2G) and the fat mass percentage (Figure 2I), compared to the control group. These data suggested that 8-week FE is an effective exercise modality for the middle-aged and elderly women to improve their body composition.

**Figure 2:**
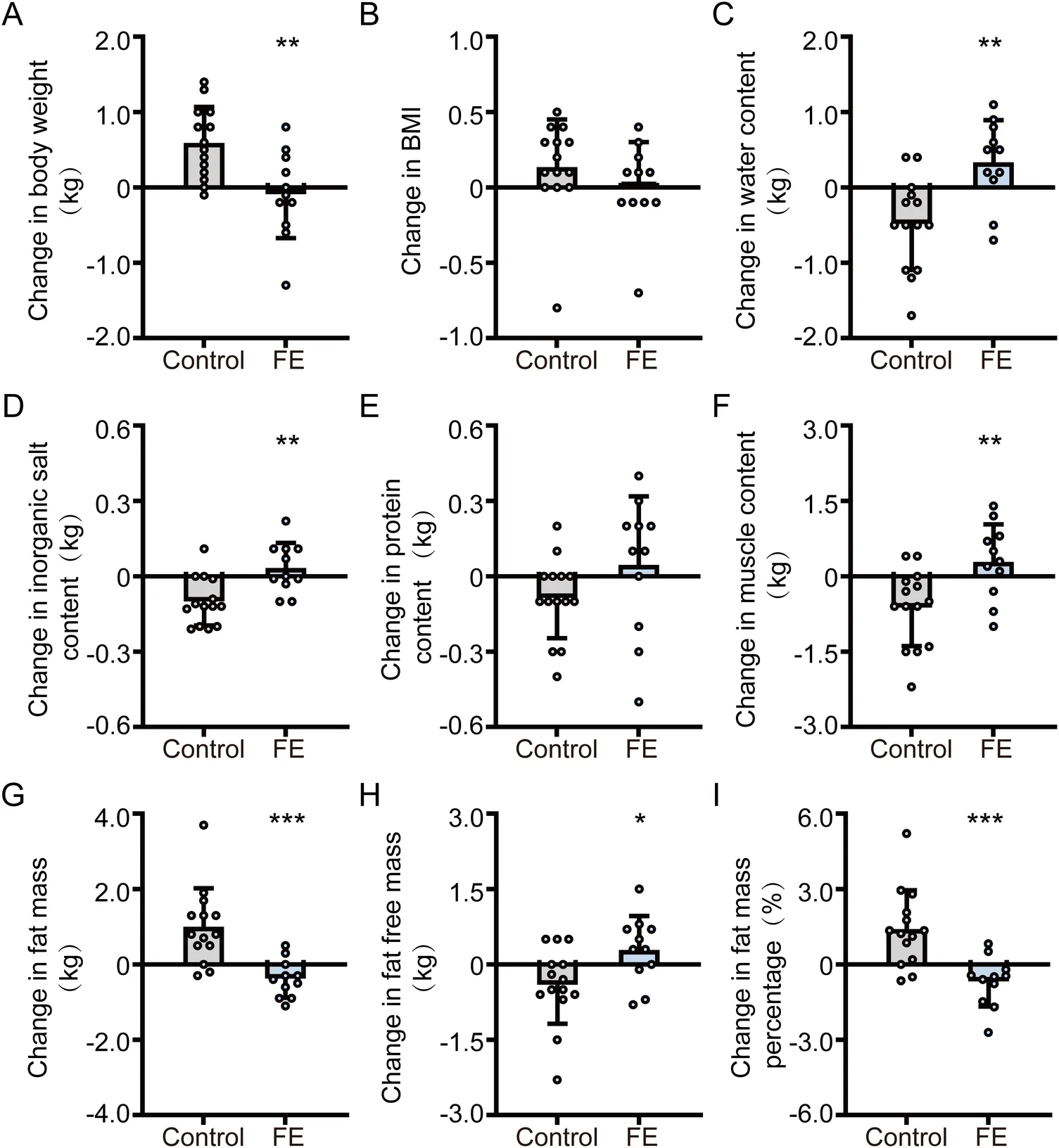
Effects of 8-week FE on the body composition in middle-aged and elderly women. Statistical data showing the changes of the **(A)** body weight, **(B)** BMI, **(C)** water content, **(D)** inorganic salt content, **(E)** protein content, **(F)** muscle content, **(G)** fat mass, **(H)** fat-free mass, and **(I)** fat mass percentage after 8-weeks FE (*n* = 11–15). *P *<* 0.05, **P *<* 0.01, ***P *<* 0.001 vs. the Control group. The data are means ± S.D.

### FE improved central hemodynamics and arterial stiffness

CVD is one of major health risk factors for the middle-aged and elderly women, which could be prevented or improved by regular exercise. The present study demonstrated that HR (Figure 3A), central arterial blood pressure (Figures 3B,C), and AIx (Figure 3D), in middle-aged and elderly women decreased after 8-week FE. However, these parameters of the control group were relatively increased to a certain extent. In addition, no significant difference in SEVR and and AIx@75 were detected between the FE group and the control group (Figures 3E,F). These data indicated that 8-week FE improved central hemodynamics in middle-aged and elderly women.

**Figure 3:**
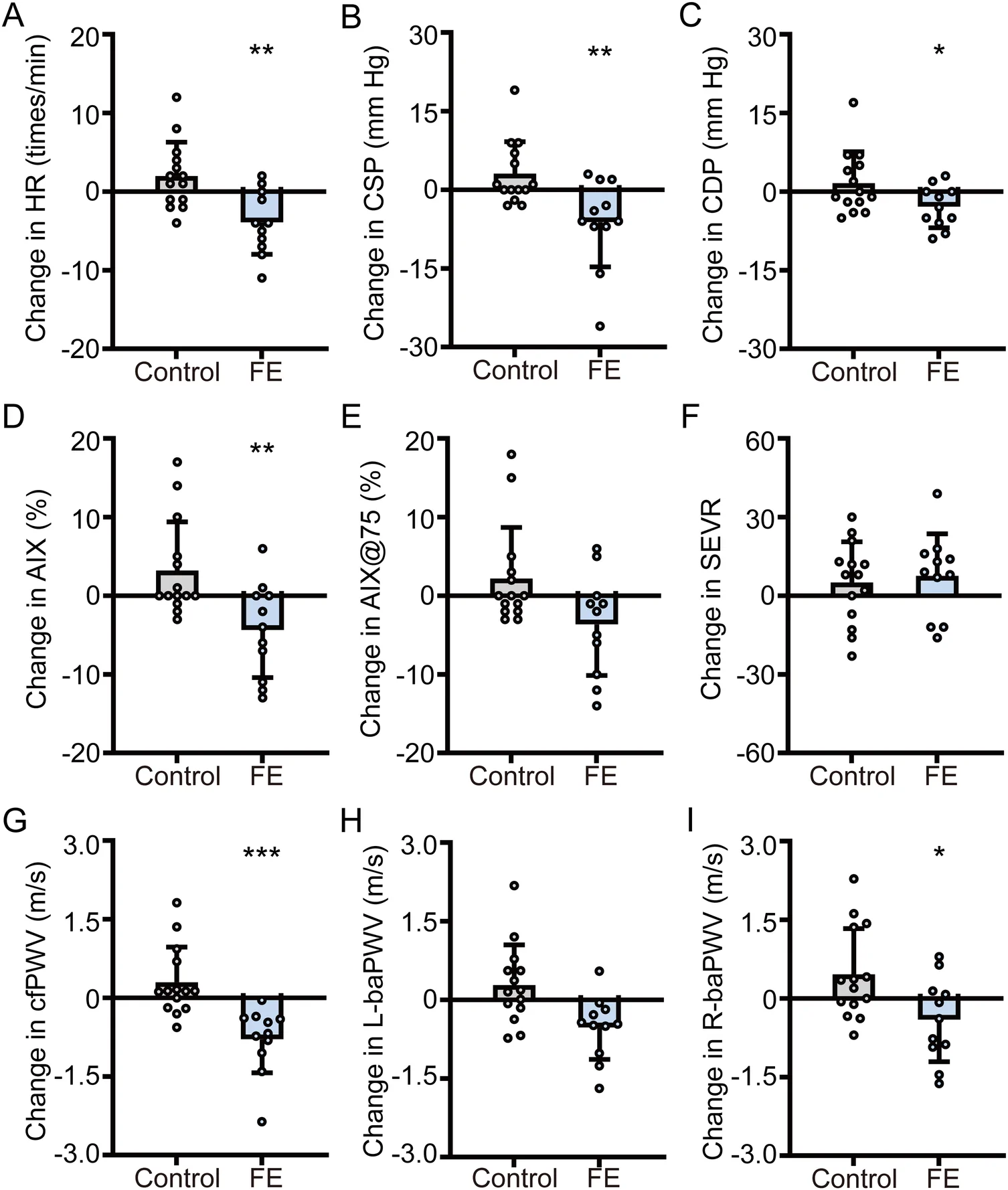
Effects of 8-week FE on central hemodynamic parameters and arterial stiffness in middle-aged and elderly women. Statistical data showing the changes in the **(A)** HR, **(B)** CSP, **(C)** CDP, **(D)** AIx, **(E)** AIx@75, **(F)** SEVR, **(G)** cfPWVR, **(H)** L-baPWV, and **(I)** R-baPWV after 8-weeks FE (*n* = 11–15). *P *<* 0.05, **P *<* 0.01, ***P *<* 0.001 vs. the Control group. The data are means ± S.D.

As is known, increased arterial stiffness is closely associated with the elevation of blood pressure, accompanied with the occurrence and ongoing process of cardiovascular and cerebrovascular diseases. Here, we observed that both of cfPWV and R-baPWV decreased after 8-week FE (Figures 3G–I). These data indicated that 8-week FE is an effective way to prevent the increase of arterial stiffness and the subsequent occurrence of hypertension.

### FE enhanced cardiac autonomic function

Furthermore, we found that HRV appeared to be on the rise after 8-week FE compared to the control group (Figure 4A). The analysis of the time-domain of HRV indicated that there was no inter-group difference between RMSSD and pNN50 (Figures 4B,C). However, SDNN and NN50 significantly increased in the FE group compared to the control group (Figures 4D,E). Meanwhile, the analysis of the frequency-domain of HRV showed that LF significantly decreased (Figure 4F), while HF significantly increased (Figure 4G) in the FE group compared to the control group. In consistency with these results, LF/HF significantly decreased (Figure 4H). In addition, an increasing trend occurred in TP of the FE group (Figure 4I). These data suggested that 8-week FE could effectively improve the cardiac autonomic function in middle-aged and elderly women by intensifying the parasympathetic activity and reducing the sympathetic activity.

**Figure 4:**
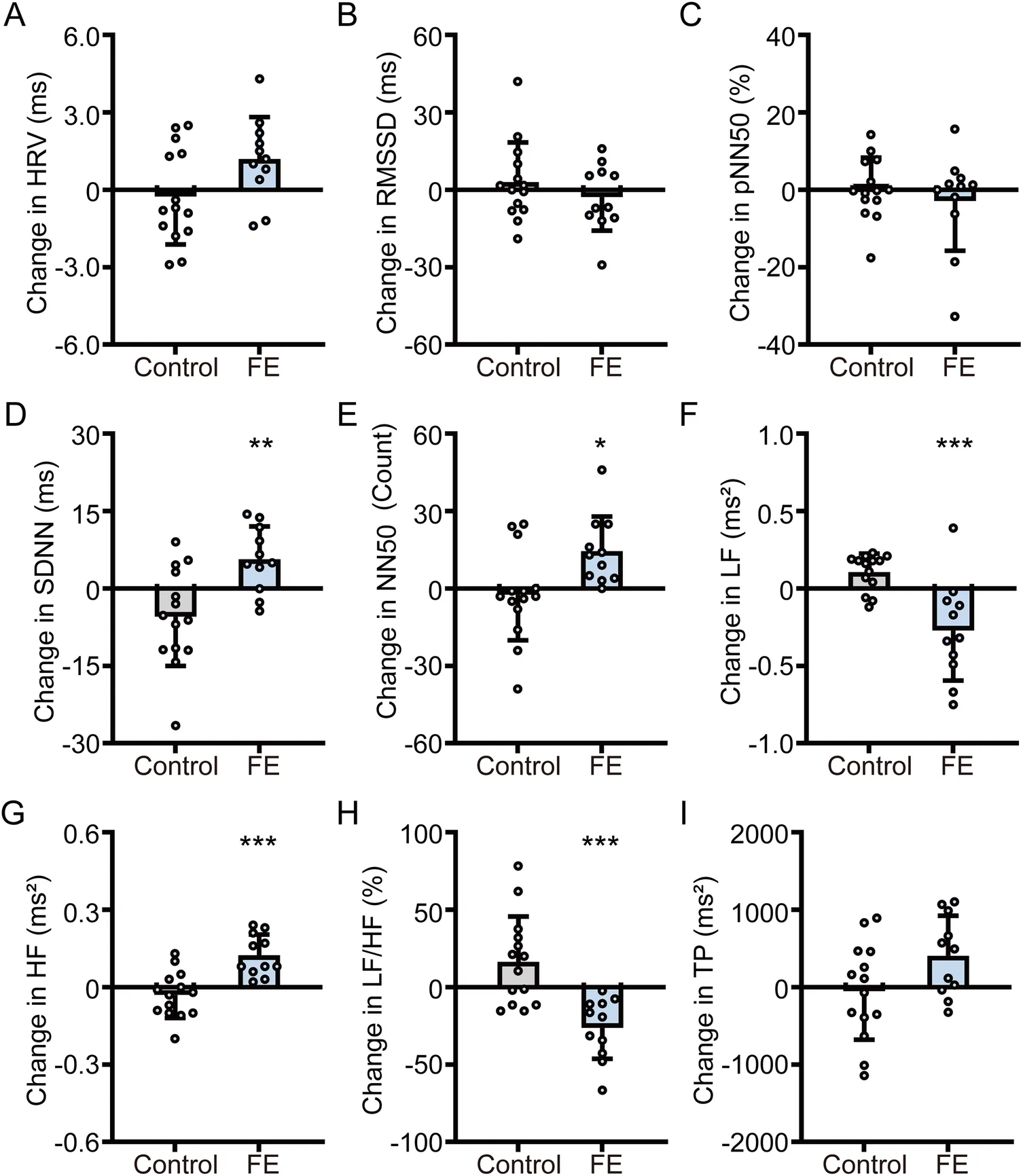
Effects of 8-week FE on cardiac autonomic function in middle-aged and elderly women. Statistical data showing the changes in the **(A)** HRV, **(B)** RMSSD, **(C)** pNN50, **(D)** SDNN, **(E)** NN50, **(F)** LF, **(G)** HF, **(H)** LF/HF, and **(I)** TP after 8-weeks FE (*n* = 11–15). *P *<* 0.05, **P *<* 0.01, ***P *<* 0.001 vs. the Control group. The data are means ± S.D.

### FE altered gut microbial composition and improve cardiovascular function

Accumulating studies suggested that gut microbiome is closely related to the cardiovascular health, and increased *Firmicutes*/*Bacteroidetes* (F/B) ratio has been proved as an indicator of CVD (24). As illustrated in Figure 5A, thePie charts visually suggested a trend of higher *Bacteroidota* and *Euryarchaeota* and lower *Firmicutes* abundance in the FE group post-intervention. Meanwhile, the weighted principal coordinate analysis (PCoA) revealed a descriptive visual clustering trendbetween the FE-pre and FE-post group (Figure 5B). Relevant Bray-Curtis Adonis results are provided in Table 3. Besides, our sequencing and bioinformatics analysis revealed that the abundance of *Bacteroidota* displayed a strong correlation with the change in baPWV, HRV and HF; the abundance of *Firmicutes* displayed a strong correlation with the change in baPWV, and cfPWV (Figure 5C). In addition, we observed the abundance of *Euryarchaeota* had a strong correlation with the changes in TP, SDNN, HF, and LF. The changes in autonomic function indices, such as NN50, PNN50, HF, and LF were associated with the abundance of *Acidobacteriota* and *Synergistota* (Figure 5C). However, no difference of the *Acidobacteriota* and *Synergistota* abundance were obeserved between the FE group and the FC group (Figure 5A). These data implied that the elevated *Bacteroidota* and *Euryarchaeota* abundance, as well as the declined *Firmicutes* abundance maybe associated with the improvement of cardiovascular function in middle-aged and elderly women after 8-week FE.

**Figure 5:**
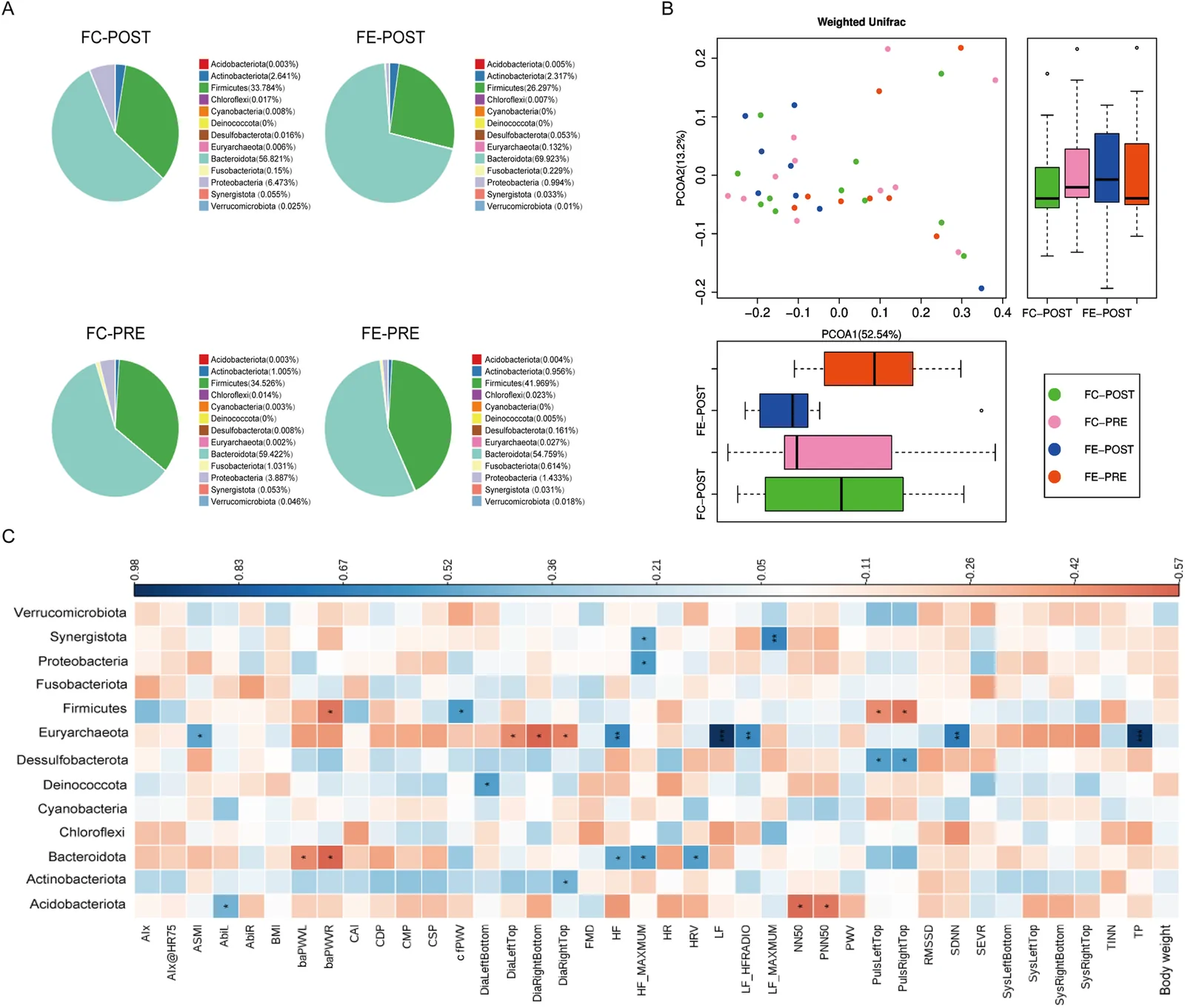
8-week FE altered gut microbial composition in middle-aged and elderly women. **(A)** Phylum comparison for the abundance of gut microbiota between the control group and the FE group. **(B)** Principal coordinate plot (PCoA) for *β*-diversity showing the clustering of gut microbial communities in the control group and the FE group after 8-week intervention. **(C)** The correlations between changes in cardiovascular function parameters and changes in gut microbial abundance,these exploratory correlation analyses were not corrected for multiple comparisons and should be interpreted cautiously given the small sample size. *P *<* 0.05, **P *<* 0.01, ***P *<* 0.001.

**Table 3:** Pair-wise Adonis (PERMANOVA, Bray-Curtis distance) results for bacterial community comparisons.

Group	df	SumsOfSqs	MeanSqs	F.Model	R2	Pr(>F) (*P*-value)
FC-POST vs. FC-PRE	1	0.12	0.12	0.41	0.02	0.97
FC-POST vs. FE-POST	1	0.40	0.40	1.59	0.08	0.04
FC-PRE vs. FE-PRE	1	0.34	0.34	1.25	0.07	0.2
FE-POST vs. FE-PRE	1	0.15	0.15	0.59	0.04	0.91

### FE increase SCFAs levels and modulates serum vascular aging biomarkers

In order to uncover the underlying molecular mechanisms, the vascular aging biomarkers and SCFAs levels were evaluated. In the present study, we found that 8-week FE significantly upregulated serum eNOS level (Figure 6A) and downregulated ICAM-1 level (Figure 6C), compared to the control group. And, no significant differences in TNF-α, VEGF, and VCAM-1 levels were detected between the FE group and the control group (Figures 6B–E), despite a decrease trendence in TNF-α level was observed in the FE group. Besides, we demonstrated that these biomarkers, including eNOS, ICAM-1, VCAM-1, and TNF-α, were strongly associated with the abundance of *Bacteroidota*, *Euryarchaeota*, and *Deinococcota* (Figure 6F). These data suggested that FE-influenced gut microbial changes might be involved in the regulation of the expression levels of these vascular aging biomarkers.

**Figure 6:**
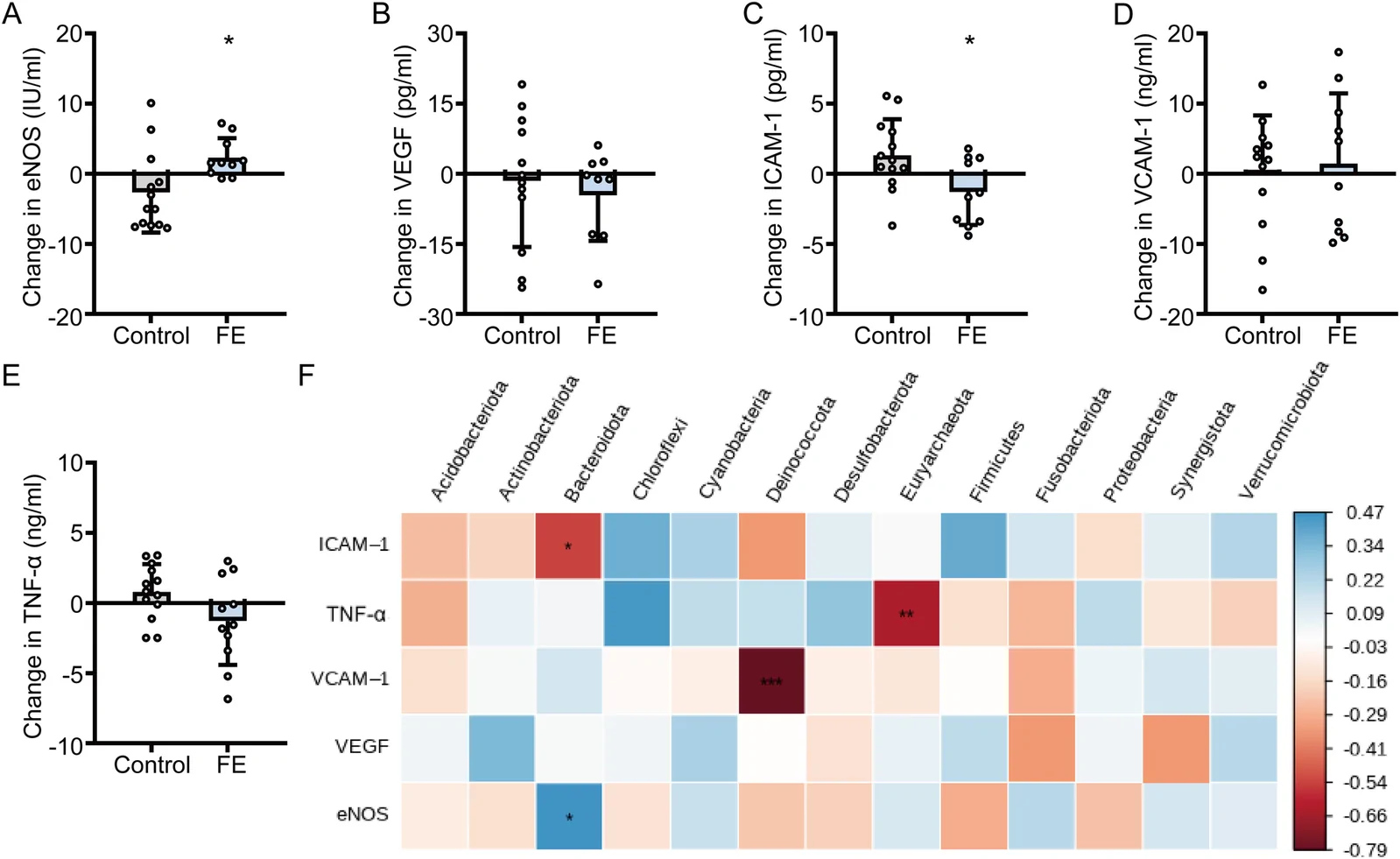
Effects of 8-week FE on vascular aging biomarkers in middle-aged and elderly women. Statistical data showing the changes in the **(A)** eNOS, **(B)** VEGF, **(C)** ICAM-1, **(D)** VCAM-1, and **(E)** TNF-α in the serum of the middle-aged and elderly women (*n* = 11–15). **(F)** The correlations between the changes in vascular aging biomarkers and the changes in gut microbial abundance of the middle-aged and elderly women, these exploratory correlation analyses were not corrected for multiple comparisons and should be interpreted cautiously given the small sample size. *P *<* 0.05, **P *<* 0.01, ***P *<* 0.001. The data are means ± S.D.

As is known, an unbalance of SCFAs production by intestinal bacteria is one of risk factors of CVD (25). Here, we observed that 8-week FE significantly elevated the levels of acetic acid, propionic acid, and caproic acid in the fecal samples of middle-aged and elderly women (Figures 7A,B,G). However, no significant changes in butyric acid, isovaleric acid, isobutyric acid, and valeric acid were detected between the FE group and the control group (Figures 7C–F). Meanwhile, our data suggested that the abundance of *Bacteroidota* and *Desulfobacterota* have a strongly association with the levels of acetic acid, isovaleric acid, and the abundance of *Euryarchaeota* have a strongly association with the level of caproic acid (Figure 7H).

**Figure 7:**
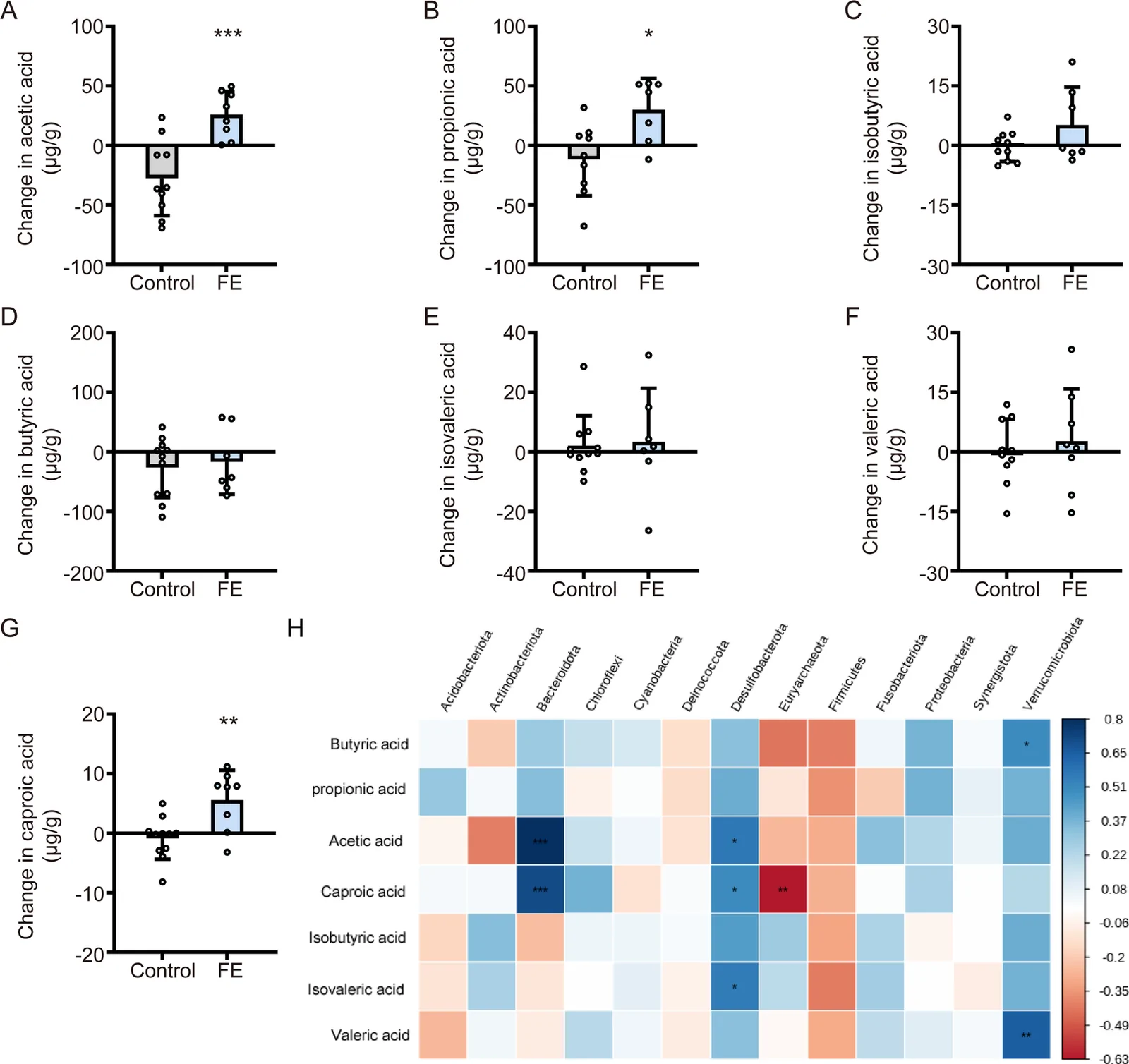
Effects of 8-week FE on fecal SCFAs in middle-aged and elderly women. Statistical data showing the changes in the **(A)** acetic acid, **(B)** propionic acid, **(C)** isbutyric acid, **(D)** butyric acid, **(E)** isovaleric acid, **(F)** valeric acid**,** and **(G)** caproic acid level in the fecal samples of the middle-aged and elderly women (*n* = 11–15). **(H)** The correlations between the changes in fecal SCFAs levels and the changes in gut microbial abundance of the middle-aged and elderly women, these exploratory correlation analyses were not corrected for multiple comparisons and should be interpreted cautiously given the small sample size. *P *<* 0.05, **P *<* 0.01, ***P *<* 0.001. The data are means ± S.D.

## Discussion

The adverse changes of arterial stiffness, hemodynamics, and cardiovascular autonomic modulation have been observed in the aging process of females during menopause (26). Previous studies have demonstrated that exercise training could provide cardiovascular and autonomic benefits (27). In the present study, our data suggested that 8-week FE could improve the body composition, andcentral hemodynamic parameters, prevent the increase of arterial stiffness and the subsequent occurrence of hypertension, and enhance cardiac autonomic function by intensifying parasympathetic activity and reducing the sympathetic activity in middle-aged and elderly women. These cardiovascular benefits might be correlated with the improvement in gut microbial composition after 8-week FE intervention, which elevate the SCFAs levels and alter serum vascular aging biomarker levels and thus enhance endothelial function. Nevertheless, formal mediation analysis could not be performed in the present study due to the limited sample size, meaning no definitive causal pathways can be confirmed.

Menopause, the most significant milestone in women's health, results in unfavorable changes in multiple health risk factors, such as increased fat mass, decreased lean mass, and high blood pressure, which further threaten their quality of life (28–30). In contrast, lifestyle modifications such as diet and exercise interventions, including aerobic exercise, high-intensity interval training, and mind-body exercise, has been proven to effectively improve cardio-metabolic health and cognitive function and thus improve the quality of life in middle-aged and elderly people (31–35). Some studies have reported that moderate physical exercise during the menopause transition and postmenopausal period could prevent the accumulation of body fat (36, 37). Vibration exercise, a relatively safe exercise modality for aged people, has also been demonstrated to enhance muscle activity and improve cardiovascular function (38). Consistent with these observations, we detected that 8-week FE prevented the accumulation of fat mass, and the loss of water content, inorganic ion content, muscle content, and fat free mass. These results indicated that 8-week FE effectively alleviates body composition deterioration, which may in turn reduce cardiovascular health risks in middle-aged and elderly women.

Over the past decades, the beneficial effects of exercise on cardiovascular function have been well documented. A recent research demonstrated that acute small- and large-amplitude FE imposed no extra threats to cardiovascular stress (21), suggesting that FE might be a safe and suitable exercise modality for middle-aged and elderly people to improve their cardiovascular health. Notably, our study is the first to demonstrate that 8-week FE could significantly decrease HR, CSP, CDP, AIx, cfPWV, and R-baPWV as compared to the control group. These data confirmed that long-term FE could improve central hemodynamic parameters and prevent the potential increase in arterial stiffness, and thus effectively alleviate the incidence of CVD in middle-aged and elderly women. Meanwhile, our data also highlight FE's beneficial effects on the autonomic nervous system, a key regulator of cardiovascular circulation and metabolism (39). The sympathetic overactivity is a well-established risk factor for CVD, such as hypertension and heart failure (40). It has been reported that vibration exercise could facilitate the decrease of cardiac sympathetic nerve activity and promote parasympathetic nerve activity (41, 42). Similarly, we found that FE lowered sympathetic activity and elevated parasympathetic activity, which may be attributed to FE's ability to reduce sympathoexcitation by inhibiting neuronal activity in brain regions that regulate cardiovascular function (43, 44). However, the neurobiological mechanisms underlying long-term FE's cardiovascular benefits require further investigation, especially considering the emerging role of the gut-heart axis (a bidirectional communication pathway between the gut microbiota and cardiovascular system) in mediating exercise-induced cardiovascular protection.

Recently, a growing body of evidence links adverse gut microbiota alterations, such as increased F/B ratio, to microbial dysbiosis and dysfunction in the cardiovascular system, autonomic nerve activity, immune, and endocrine system (45–47). These studies established that the gut-heart axis bridges gut microbiota composition/diversity with autonomic nerve activity. Additionally, exercise intervention has been reported to downregulate the F/B ratio in both mouse models and humans (48–50), suggesting exercise may act as a “modulator” of the gut-heart axis to improve cardiovascular health. Consistent with these studies, our results showed that 8-week FE reshaped the overall gut microbial composition in middle-aged and elderly women. Moreover, we found that the decrease in PWV, a gold-standard marker of arterial stiffness, correlated with increased *Bacteroidota* abundance and decreased *Firmicutes* abundance. This preliminary study observed associations between FE-altered levels of *Bacteroidota* and *Firmicutes* and alleviated arterial stiffness in middle-aged and elderly women Furthermore, we also found that the changes in *Acidobacteriota* and *Dessulfobecterota* correlated with central hemodynamic parameters, while *Bacteroidota*, *Proteobacteria*, *Acidobacteriota,* and Euryarchaeota abundance correlated with cardiac autonomic function—another key node of the gut-heart axis. Notably, it was reported that *Euryarchaeota* was positively associated with CA-125, a promising biomarker in some cardiovascular diseases (51). In our study, we found that 8-week FE increased *Euryarchaeota* abundance, indicating the cardiovascular benefit of 8-week FE. Collectively, these data strongly suggest that FE improves cardiovascular function in middle-aged and elderly women at least in part by reshaping gut microbiota composition and restoring gut-heart axis homeostasis, providing a novel mechanistic link between local vibration exercise, gut health, and cardiovascular protection.

Meanwhile, our measurements of vascular aging biomarkers further supported FE's cardiovascular benefits. We found that ICAM-1 was downregulated and eNOS was upregulated after 8-week FE intervention. Critically, changes in both ICAM-1 and eNOS expression correlated with the abundance of *Bacteroidota*, a founding coincidence with the previous studies (52, 53). This suggests that 8-week FE may modulate vascular health in middle-aged and elderly women by reshaping *Bacteroidota* abundance to regulate ICAM-1 and eNOS expression, thereby directly promoting cardiovascular function.

Gut microbes are involved in the synthesis and transformation of a series of bioactive metabolites, such as SCFAs, trimethylamine oxide (TMAO), amino acids, vitamins, bile acids, toxins, anthocyanins, and phytoestrogens, which can be used as substrates or signaling molecules (54). Increasing evidence suggests that SCFAs, which could enter the blood circulation through intestinal epithelial cells and act on specific receptors in cardiovascular system, thereby regulate cardiovascular function (55). The reduction of SCFAs level promotes the transfer of endotoxin to the blood circulation, resulting in cardiovascular dysfunction (56). Notably, exercise has been shown to enhance gut microbial diversity, and this shift is closely tied to elevated SCFA production, ultimately contributing to improved cardiovascular function (19). The mechanistic link between SCFAs and vascular protection is further clarified by evidence that acetate (a major SCFA) activates G-protein-coupled receptors on vascular endothelial cells, triggering nitric oxide (NO) release and reducing arterial stiffness (55). This aligns with our earlier observation of eNOS upregulation after 8-week FE intervention, strongly suggesting SCFAs act as upstream regulators of endothelial NO synthesis in our study. Moreover, increased acetic and propionic acid levels are known to improve endothelial function and mitigate systemic inflammation, further supporting SCFAs' role in cardiovascular protection. In line with these insights, we demonstrated that 8-week FE significantly increased acetic acid, propionic acid, and caproic acid levels in fecal samples. We also identified specific microbe-SCFA associations. Our data showed that in the acetic acid and caproic acid levels were associated with *Bacteroidota* abundance. The acetic acid, caproic acid, and isovaleric acid levels were associated with *Dessulfobecterota* abundance. And the caproic acid level was associated with *Euryarchaeota* abundance. These results confirm that FE-induced SCFA elevation is not random but driven by targeted reshaping of specific gut microbial phyla. However, it should be noticed that the large set of exploratory Spearman correlations among microbial taxa, cardiovascular indices, biomarkers and SCFAs was performed on a small sample. These correlation coefficients are potentially unstable, and accordingly, these findings are hypothesis-generating rather than robust confirmatory associations.

## Conclusions

In conclusion, this study confirms 8-week FE enhanced parasympathetic activity, reduced sympathetic activity, thereby alleviating arterial stiffness and thus improves cardiovascular function in middle-aged and elderly women. Concurrent shifts in gut microbiota composition, increased key SCFA concentrations level, and favorable changes in vascular biomarkers, including eNOS upregulation, ICAM-1 downregulation. This work identifies correlational associations between FE and gut-heart axis-related indicators, suggesting FE may serve as a safe intervention to support cardiovascular health in middle-aged and elderly women. This study also have several limitations. First, dietary intake was not systematically recorded, and we did not collect participants' menopausal status or smoking information, which may introduce confounding influences on gut microbiota and cardiovascular measurements. Second, the lack of a sham control group means we cannot fully rule out potential psychological expectancy effects of exercise participation. Third, all four drop-outs occurred only in the FE group, which may introduce selection bias that could distort intervention-effect estimates, and ITT imputation was not feasible given the lack of post-intervention data for withdrawn participants. Additionally, our correlational analyses cannot establish causal relationships, and the sample of middle-aged women restricts the generalizability of our results to advanced elderly populations. Collectively, these data show correlational associations between FE-related alterations in gut microbial profiles and cardiovascular-related indices; causal gut-heart mechanistic inferences cannot be drawn given the study limitations and exploratory nature of these analyses. Thus, further well-controlled trials are warranted to validate our gut-heart axis findings.

## Data Availability

The original contributions presented in the study are publicly available. This data can be found here: [https://doi.org/10.57760/sciencedb.38965].
