# Supplementary material for: Eight-week flexi-bar vibration exercise improves cardiovascular function accompanied by altered gut microbiota and metabolites in middle-aged and elderly women: a randomized controlled trial
Source: Front Cardiovasc Med. 2026 Oct 27;13:1925079. doi: 10.3389/fcvm.2026.1925079 (PMC13748288; doi:10.3389/fcvm.2026.1925079)
Supplement: Supplementary file 1 [file Table1.docx]

**Table 1 The physical conditions of the participants at the beginning of the study**

| Parameters | Control group | FE group |
| --- | --- | --- |
| Age (year) | 55.47 ± 5.45 | 56.53 ± 5.64 |
| Height (cm) | 158.53 ± 6.08 | 158.93 ± 4.79 |
| Weight (kg) | 57.02 ± 5.67 | 55.60 ± 6.22 |
| BMI (kg/m^2^) | 22.71 ± 2.16 | 22.02 ± 1.86 |
| Water content (kg) | 27.99 ± 2.85 | 26.77 ± 2.22 |
| Inorganic salt content (kg) | 2.67 ± 0.28 | 2.60 ± 0.21 |
| Protein content (kg) | 7.44 ± 0.72 | 7.13 ± 0.61 |
| Muscle content (kg) | 35.92 ± 3.63 | 34.35 ± 2.84 |
| Fat mass (kg) | 18.59 ± 3.44 | 18.91 ± 4.18 |
| Fat-free mass (kg) | 38.11 ± 3.84 | 36.24 ±3.15 |
| Fat mass percentage (%) | 32.65 ± 4 | 33.98 ± 4.85 |
| HR (bpm) | 60.64 ± 7.48 | 61.27 ± 6.65 |
| CSP (mmHg) | 119.79 ± 10.2 | 122.55 ± 17.03 |
| CDP (mmHg) | 75.93 ± 6.87 | 79.73 ± 7.89 |
| cfPWV (m/s) | 7.16 ± 0.68 | 7.3 ± 0.65 |
| L-baPWV (m/s) | 11.3 ± 0.82 | 11.84 ± 1.25 |
| R-baPWV (m/s) | 11.18 ± 0.8 | 11.22 ± 1.19 |
| AIX (%) | 37.07 ± 10.81 | 35.73 ± 9.68 |
| AIX@75 (%) | 29.86 ± 9.84 | 30.55 ± 8.55 |
| HRV (m/s) | 7.27 ± 1.97 | 7.25 ± 2.55 |
| RMSSD (m/s) | 29.19 ± 19.57 | 32.32 ± 18.43 |
| pNN50 (m/s) | 9.59 ± 18.87 | 11.98 ± 18.95 |
| SDNN (m/s) | 35.84 ± 13.84 | 35.76 ± 12.62 |
| NN50 (m/s) | 23.93 ± 44.28 | 27.73 ± 40.43 |
| HF | 0.23 ± 0.06 | 0.24 ± 0.06 |
| LF/HF | 1.19 ± 1.24 | 1.45 ± 1.14 |
| TP (ms^2^) | 936.79 ± 787.79 | 954 ± 809.78 |
